# Flexibility of habitat use in novel environments: insights from a translocation experiment with lesser black-backed gulls

**DOI:** 10.1098/rsos.160164

**Published:** 2017-01-18

**Authors:** Mariëlle L. van Toor, Elena Arriero, Richard A. Holland, Markku J. Huttunen, Risto Juvaste, Inge Müller, Kasper Thorup, Martin Wikelski, Kamran Safi

**Affiliations:** 1 Department of Migration and Immuno-Ecology, Max Planck Institute for Ornithology, Radolfzell, Germany; 2 Department of Biology, University of Konstanz, Konstanz, Germany; 3 Department of Zoology and Physical Anthropology, University Complutense of Madrid, Madrid, Spain; 4 School of Biological Sciences, University of Bangor, Bangor, UK; 5 School of Forest Sciences, Faculty of Science and Forestry, University of Eastern Finland, Joensuu, Finland; 6 North Karelia University of Applied Sciences, Joensuu, Finland (retired); 7 Department of Biology, University of Turku, Turku, Finland; 8 Center for Macroecology, Evolution and Climate, University of Copenhagen, Copenhagen, Denmark

**Keywords:** ecological specialization, flexibility, habitat use, niche comparison, species distributionmodel, translocation

## Abstract

Being faced with unknown environments is a concomitant challenge of species' range expansions. Strategies to cope with this challenge include the adaptation to local conditions and a flexibility in resource exploitation. The gulls of the *Larus argentatus-fuscus-cachinnans* group form a system in which ecological flexibility might have enabled them to expand their range considerably, and to colonize urban environments. However, on a population level both flexibility and local adaptation lead to signatures of differential habitat use in different environments, and these processes are not easily distinguished. Using the lesser black-backed gull (*Larus fuscus*) as a system, we put both flexibility and local adaptation to a test. We compare habitat use between two spatially separated populations, and use a translocation experiment during which individuals were released into novel environment. The experiment revealed that on a population-level flexibility best explains the differences in habitat use between the two populations. We think that our results suggest that the range expansion and huge success of this species complex could be a result of its broad ecological niche and flexibility in the exploitation of resources. However, this also advises caution when using species distribution models to extrapolate habitat use across space.

## 1. Background

The ability to cope with the challenges of finding resources under changing conditions, caused, for example, by environmental change, range expansion into novel environments or changes in competition, can impact the survival and reproductive success of individuals directly. One strategy to cope with such situations is the flexibility in how available habitat is used, and which resources the individuals of a species or populations specialize in (e.g. [1]). While ecological specialists might benefit from a higher relative fitness under favourable conditions, theory predicts that generalist species, or species with a flexibility in habitat use, should have a higher ability to deal with unfamiliar and/or stochastically changing environments (e.g. [2]). As a consequence, generalist species might also be pre-adapted for the colonization of novel environments. Indeed, it has been shown that dietary flexibility and the ability to exploit novel food resources are related to the success of species invasions and the colonization of anthropogenic habitats [3–8]. Consequently, ecological flexibility is an important trait to consider for species that are currently shifting or expanding their range into formerly unoccupied habitat [2].

One group of species whose success of colonizing novel habitats has been attributed to ecological flexibility are the large white-headed gulls of the *Larus argentatus-fuscus-cachinnans* group, a species complex with a circumpolar distribution in the Northern hemisphere. Species of this complex, for example, the lesser black-backed gull (*Larus fuscus*, L. 1758), do not only readily use resources made accessible through human activities [9–11], but are also in the process of becoming invasive [12, 13]. Furthermore, genetic analyses have revealed that this complex has undergone a very recent range expansion and an overall population growth [14, 15]. These findings are indicative of a high degree of ecological flexibility (see also [5]). This flexibility in habitat use might thus underlie the ability of the individuals to exploit different resources in different environments. The same individual can therefore occupy different realized niches, as part of a larger fundamental niche, when being confronted with spatially distinct resource distributions.

However, differential habitat use between populations is an ambiguous signature that can also be caused by processes other than ecological flexibility. In the presence of restricted gene flow between populations, differences in habitat use between populations could represent local adaptation as a consequence of a divergence in ecological niches owing to natural selection. According to Kawecki & Ebert [16], local adaptation can arise when divergent selection acts on the habitat preferences of local populations, leading to a fitness advantage in conditions resembling their local original environment. The requirement for local adaptation to occur is, among others, restricted gene flow. And in fact, previous studies suggested that the *Larus argentatus-fuscus-cachinnans* group form a ring species [17], with low levels of hybridization between what were considered sub-species [18–20], even in areas of direct spatial contact. These findings were supported by the observation of consistent individual differences in resource use even within a population [21–24]. Thanks to recent genetic analysis [25], the ring species hypothesis is now largely disregarded and the taxonomy of the species complex is being reorganized [25, 26]. Yet, the claims of local adaptation and niche divergence between the different populations remain seemingly in conflict with the more recently postulated high ecological flexibility in the species. Both ecological flexibility as well as local adaptation can result in differential habitat use on a population level. Consequently, distinguishing between differences in habitat use owing to either ecological flexibility or adaptation to locally available resources is not easy, particularly when habitat use of the same individuals under different environmental contexts remains unknown.

While the patterns of flexibility and specialization are similar between individuals of different populations, the underlying processes are fundamentally different. Observed differences in habitat use can reflect different realized niches owing to the different availability of habitat resources, or the manifestations of mutually exclusive fundamental niches eventually defining ecologically distinct (sub-)species. Therefore, we think that it is elementary to consider and distinguish between ecological flexibility and local adaptation as potentially exclusive explanations for differential habitat use when the volume of the fundamental niche of a species is unknown. From a practical perspective, not distinguishing between and accounting for the different processes will limit the range of conclusions that can be drawn from studying a species' habitat use. Especially studies that only focus on a limited part of the annual cycle and/or are a non-representative sample of the population might not uncover the entirety of the fundamental ecological niche, and thus underestimate the breadth of resources and habitats individuals of a species might be able to exploit. On the contrary, neglecting the existence of local adaptation can lead to the overestimation of the ability of a species to cope with changing conditions. Thus, not accounting for either process can have stark consequences for the interpretation of observed differences in habitat use between populations, or species, but also affects the interpretation of predicted distributions of suitable habitat based on species distribution modelling. As species distribution models are frequently used in the context of conservation planning (e.g. [27, 28]) or in predicting the spread of invasive species (e.g. [29]), both ecological flexibility and local adaptation limit the transferability of obtained results [30–33].

In this study, we put the two contrasting mechanisms translating to a signature of differential habitat use on a population level to a test. Here, we use location data of individuals from two spatially separate populations of lesser black-backed gulls (*L. f. fuscus*), caught in Southern Finland and on Solovki Island in Russia. These data were collected using global positioning system (GPS) and were available to us from a previous study focusing on navigation in this species [34, 35]. We first aim to identify whether differential habitat use can be observed between populations. We compare habitat use between individuals of the two populations using species distribution models, expecting to find differential habitat use that is indicative of either mechanism. We then distinguish between adaptation to local conditions and flexibility by investigating whether and how individuals use habitat differently when confronted with an unknown environment based on a translocation experiment. Under the scenario of adaptation to local conditions, we expect habitat use after translocation to be similar to habitat use at the site of origin, after correcting for differential availability of resources between sites. Ecological flexibility, however, should lead to habitat use that is different from the predictions based on the native population. For the translocation, the individuals were caught in two populations and were translocated to unfamiliar sites. Individuals caught in Finland were released on Helgoland, where a different subspecies breeds in high numbers, whereas individuals caught on Solovki Island were brought to Kazan which is outside the species breeding range. Using these data, we compared niche overlap both between individuals within populations as well as between populations, and thus assessed the degree of specialization and ecological divergence.

We put the potential differences in resource use into the context of the differences in the habitats by comparing control and translocated individuals, which should provide insight into how differentiated habitat use might be across space, and unravel the underlying process. In addition to this population-level comparison, we also explore potential differences in habitat use between individuals of the same population. Owing to the previously described differences in resource use even within a population [21–23], we expect that the tagged individuals show some differences in habitat use within treatment groups.

## 2. Methods

### 2.1. Tracking data

The original tracking data used for this study were published in a previous study [34] and are available from the Movebank Data Repository http://www.datarepository.movebank.org [35]. This original dataset, however, also contains data from individuals which received treatments in addition to translocation. Those individuals were not considered in this study.

Adult lesser black-backed gulls (*L. f. fuscus*) were caught at two different locations in Southern Finland (between 23 E 64 N and 30 E 61 N, hereafter referred to as ‘Finland’) and on Solovki Island in the White Sea (36 E 65 N, hereafter ‘Solovki Island’) in the year 2009 (for more details, see [34]). All individuals were equipped with solar-powered GPS tags (Microwave Telemetry, Inc., MD, USA) using Teflon harnesses. The Finnish control animals were caught during the breeding season and released without further treatment. Birds that had been caught in the same area after the same breeding season were translocated to Helgoland (79 E 54 N, group is termed ‘Helgoland’) by plane. Likewise, the individuals caught on Solovki Island were either released, or transported to Kazan (49 E 55 N, group is termed ‘Kazan’) by plane and released there. Helgoland supports a large number of breeding pairs of two different subspecies (*L. f. intermedius* and *L. f. fuscus*), whereas the region around Kazan is a common stopover site for *L. f. fuscus* migrating south from the White Sea. Both Helgoland and Kazan sites provide foraging areas for the birds. In addition to the deployment with GPS-tags, seven of the individuals from Finland had also been subjected to an immunization treatment (diphtheria/tetanus-toxin) and were kept for up to 5 days before translocation and release. The effects of this weak immunization wore off after a few hours and we expect no effects of this weak immune challenge on the behaviour of the individuals after release (see also [36]).

A total of about 50 000 GPS-fixes for control and translocated birds had been acquired over the total duration of the study (May 2009–2011), with a mean of 3.8 GPS fixes per individual and day. Although the species is migratory, we focused the analysis only on the native breeding habitat or the release site for the translocated individuals, as individuals from both populations shared their wintering area in eastern Africa (Lake Victoria, Lake Edward, Lake Albert). We therefore filtered the data for the initial time period after release while the birds resided in the breeding areas (control birds) or in the release area (translocated birds), excluding locations below 50° latitude. Owing to the low temporal resolution of the tracking data, we could not determine the birds' behaviour when the fix was taken (e.g. using [37] or [38]) and could thus not distinguish between actual habitat use (e.g. feeding) or other behaviour (e.g. flying). For this reason, we decided to keep all locations remaining after filtering for the analyses. The final sample sizes are listed in table 1 (see also the electronic supplementary material, figure S1).

**Table 1.** Summary of the data available and used for modelling. (Here, we list the number of individuals for each capture site and treatment. The number of individuals is given as the number for which data were available, and the number originally tagged in parentheses. The locations available for modelling are the subset of the total location dataset that could be annotated with all environmental layers.)

group	treatment	release date	sample size	no. of locations for modelling
Finland	control	24 May–2 June	34 (36)	6'825
Solovki Island	control	18–19 August	20 (20)	(both groups combined)
Helgoland	translocated	16 August	12 (12)	888
Kazan	translocated	19 August	10 (10)	675

### 2.2. Displacement from the release site and start of migration

To estimate the impact of the translocation on the individuals, and the consequences that might arise for individual habitat use, we calculated the displacement for all individuals in the first 30 days post release. In addition, we compared the timing of migration of individuals in the different study groups. To determine the start of migration, we built a classifier using random forest modelling [39]. We used latitude, the cumulative and daily distance travelled as predictors for each of the locations. We evaluated the results manually by inspecting the classified trajectories.

### 2.3. General habitat use

As comparable environmental information was not available for both terrestrial and marine habitats, we restricted the application of species distribution models and the comparison of habitat use between groups to a single general habitat type. For this study, we chose terrestrial habitat, as most of the available GPS locations of birds (80.3%) were above land. This is in accordance with the literature, as lesser black-backed gulls are considered to spend a considerable amount of their foraging time on land, and in close proximity to human-associated landscape structures [9, 10]. To provide a more general overview of habitat use, however, we calculated the preference of each treatment groups for three broad habitats: terrestrial, marine and freshwater habitat. To calculate this preference, we estimated for each treatment group how often the birds were recorded in one of these habitats, and how this observation related to the availability of this habitat. To achieve this, we determined the habitat type for GPS locations with the GSHHS shoreline database [40], using only locations prior to the onset of migration. We then calculated the surface area of terrestrial, marine and freshwater habitat within the area occupied by each treatment group using convex hulls. Finally, we calculated the ratio between observed use and availability to estimate the relative use of each habitat type. Here, values close to one should indicate that the birds do not use this habitat more often than expected, and thus show neither preference nor avoidance. Values higher or lower than one, however, indicate a non-random use and therefore a preference for a certain habitat type, or respectively, its avoidance.

### 2.4. Habitat models

We chose MaxEnt [41] as our modelling framework, as it has been shown to provide good results for the general prediction of species distributions [42]. MaxEnt (short for maximum entropy) is a presence-only species distribution model that is based on a machine-learning approach. It compares the environmental conditions at presence locations with the available environment using randomly sampled background locations [41, 43]. It estimates a species' distribution by minimizing the divergence between the density of covariates at presence locations and the density of covariates at background locations. This results in a log-linear model that can contain complex interactions, and predicts the probability of presence of the species as a function of the environment [41, 43].

We initially started with 13 different remote sensing products containing a total of 75 environmental variables (table 2), including landcover, distance to sea, altitude, human impact and climatic information. When available, layers were downloaded in a resolution of 30 arc-seconds, the remaining were either interpolated to a higher resolution (anthromes, distance to sea) or reduced in resolution (GlobCover_2009) to match a 30 arc-second grid size. After preparation of the environmental variables, we annotated both the presence locations and randomly sampled background locations (see below for sample sizes) with the corresponding environmental information. Prior to the application of MaxEnt, we partitioned the data into a training dataset (75% of all presence locations) and a test dataset (the remaining 25% of the data). This allowed us to apply a twofold cross-validation for all MaxEnt models, i.e. models were first trained using the training data, and then applied to the test data to estimate the model's performance. Performance, or the model's ability to distinguish presences from background in the test data, was assessed using the area under the receiver operating curve (AUC), which is a widely used method [44] (but see [45]). It is a measure of commission (false positive) and omission (false negative) error and ranges from zero to 1, with AUC = 1 indicating perfect discrimination and AUC = 0.5 stating that the model does not perform better than random.

**Table 2.** This table lists all environmental layers used for the habitat modelling. (Also included are the sources for the different variables and the type of the variable. The contributions of the variables to the final models are listed in the electronic supplementary material, table S1.)

variable name	classification	data source
altitude	continuous	www.worldclim.org
anthromes (v1)	categorical	www.ecotope.org
bioclim (19 layers)	continuous	www.worldclim.org
distance to sea	continuous	www.ngdc.noaa.gov
terrestrial ecoregions	categorical	www.worldwildlife.org
GlobCover_2009	categorical	ionia1.esrin.esa.int
Global Lakes and Wetland Database	categorical	www.worldwildlife.org
human footprint	continuous	sedac.ciesin.columbia.edu
night-time lights	continuous	www.ngdc.noaa.gov
precipitation (12 layer)	continuous	www.worldclim.org
maximum temperature (12 layers)	continuous	www.worldclim.org
mean temperature (12 layers)	continuous	www.worldclim.org
minimum temperature (12 layers)	continuous	www.worldclim.org

First, we computed MaxEnt models for single individuals, for which we used only the presence points of individuals for which at least 25 locations were available (*n* = 62), and used 20 000 randomly sampled background locations. Using these individual MaxEnt models, we estimated the similarity of habitat use between individuals of the same treatment group using a measure of niche overlap (Bray–Curtis (BC) index, see section ‘Model comparison’ below for details). If individuals at a location were all specialized on the same habitat, this should result in high niche overlap, whereas low values of niche overlap would indicate that individuals at the same site can use different resources.

To compare habitat use between the groups, we computed MaxEnt models based on the locations of all individuals per site using 50 000 randomly sampled background locations. This resulted in one group-level model per site that incorporates the habitat use of all the individuals released at that given site. We provide spatial predictions of habitat suitability for each group-level model for the complete study area in the electronic supplementary material, figure S2.

As the control birds released in Finland were caught already during the breeding season as in contrast to the other treatment groups, we tested whether there was a difference in habitat use between breeding and post-breeding period. To do so, we calculated a MaxEnt model both for the breeding period only and both breeding and post-breeding period combined. We used these models to predict 10 000 the presence and absence locations sampled at random, and calculated the differences between the model predictions. Since the total difference summed up to 1.27%, we decided to use data for both the breeding and post-breeding period for the Finnish control birds.

### 2.5. Model comparison

To compare the predicted space use between both the individuals of a population as well as the different groups, we applied distance metrics as introduced by Warren *et al.* [46, 47]. Rödder *et al.* [48] tested the performance of a range of these indices, and from these we chose the one performing best for our application (BC index). Before comparing the predictions generated by the models, we standardized these by dividing the presence probability for each cell by the sum of the presence probability over the complete study area. Phillips *et al.* [41] as well as Rödder *et al.* [48] suggest to apply a threshold rule before comparison. Therefore, we chose to use the minimum value of the presence probability of an actual location of the training dataset of each model as a cut-off [49]. In this way, every cell having a presence probability lower than the minimum observed probability for the species was set to zero; this allowed us to only analyse those pixels of the study area for which the animals were likely to be present.

First, we calculated BC on the projections of the individual models, and calculated the niche overlap between all combinations of individuals of one site. Second, we compared habitat use between the control populations (Finland and Solovki Island) to test whether local adaptation or flexibility might occur in this subspecies of lesser black-backed gulls. Rather than comparing habitat use between each translocated group and its corresponding origin location separately, however, we pooled the data of both control groups. We did so to create a more conservative model of habitat use containing the locations of individuals from Finland as well as Solovki Island (hereafter, this group will be termed ‘control’). We then compared habitat use between the control birds and individuals translocated to Helgoland, as well as between control birds and the animals released in Kazan separately.

### 2.6. Randomizations

Without an *a priori* expectation about the amount of niche overlap under the assumption of complete sympatry, the overlap of model predictions is not biologically meaningful [46]. We resolved this problem by using randomization tests as suggested by Warren *et al.* [46, 47] (niche identity test). For each comparison (Finland–Solovki Island, control–Helgoland, control–Kazan), we ran 1000 replicates of models for the two respective groups, but with randomized group identity to simulate a shared spatial distribution. Thus, we generated an experimental distribution of expected overlap under the assumption of sympatry and compared it to the observed values. If the observed values were comparable to or higher than the expected distribution, habitat use did not differ between groups. If, however, the observed overlap of model predictions was smaller than random, the two groups were using different habitat. As the animals were released in four different locations, with two of the release sites being novel areas, the availability of habitat or resources between sites might have differed, and thus contributed to the observed differences in habitat use. To test for the contribution of differential habitat composition we ran a second set of randomizations, also with 1000 replicates each according to Warren *et al.* [46, 47] (background test). A distribution of expected differences in model predictions is generated by comparing the model of one group with the model produced for randomly placed points in the area used by the other group, simulating invariant habitat selection [46, 47]. For these models, the background environmental data had to be restricted to the area which was actually used. We did this by sampling random points within the 90% minimum convex polygons of each of the groups separately. All analyses were performed using the software MaxEnt and R [41, 50].

## 3. Results

### 3.1. Displacement from the release site and migration

**Figure 1.**
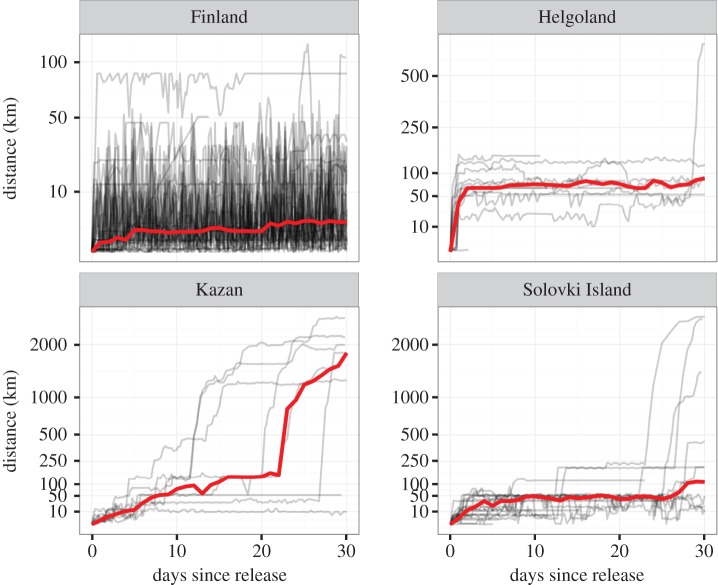
Displacement of individual gulls after release. The displacement from the site of release over the first 30 days post release. Individual birds are shown in grey, the median of the group is represented in red. Note that the actual release date differed between the groups owing to the different treatments (table 1).

**Figure 2.**
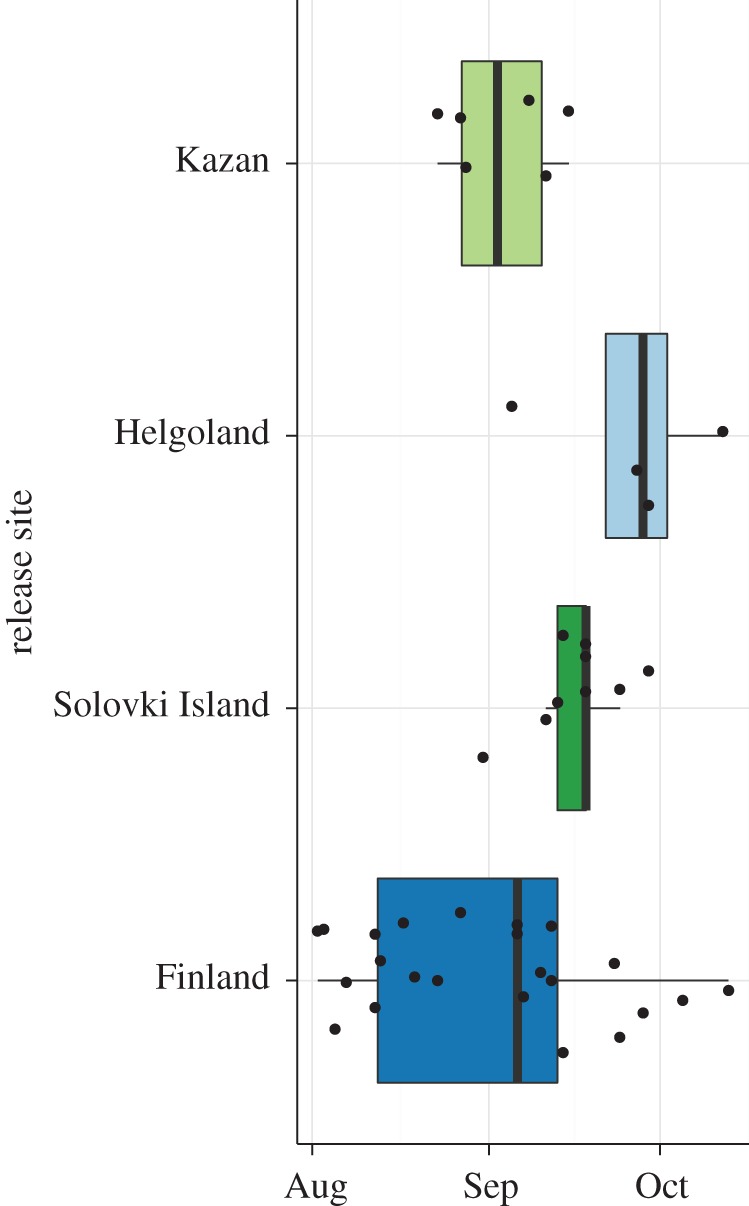
Timing of migration. The first day of migration was determined for each individual for which tracking data were available during the migratory period. The boxplot shows the distribution of the timing of autumn migration for the different groups. The boxes represent the 25%, 50% (median) and 75% quartiles. The whiskers show the 1.5-fold interquartile ranges. Black dots represent the raw data for each group.

We found that most birds stayed in the closer vicinity of the release site prior to migration. Although the birds released in Finland seemed to undertake daily trips of up to 50 km distance from the release site (figure 1), the individuals translocated to Helgoland showed an initial displacement of up to 120 km (mean = 32.0 km, s.d. = 28.96 km). One individual on Helgoland started migrating within 30 days after release. Both the individuals released on Solovki Island and Kazan showed displacement of up to 50 km from the release site, but some of them initiated migration within the first 15 days after release (figure 1). Overall, the four groups demonstrated differences in their timing of migration, and the individuals from Finland showed the greatest variability in timing (figure 2). The birds released on Helgoland initiated migration considerably later than individuals in Finland, but not significantly so (mean: 23 days, 95% confidence intervals (CIs): [−1,51] days, *p* = 0.060, Wilcoxon rank test). Finnish birds started migration significantly earlier than birds from the White Sea (mean: 15 days, 95% CIs: [1,32] days, *p* = 0.032, Wilcoxon rank test). Individuals from the White Sea started migration as the latest of all groups, and significantly later than their translocated counterparts in Kazan (mean: 14 days, 95% CIs: [3,22] days, *p* =0.018, Wilcoxon rank test).

### 3.2. General habitat use

**Figure 3.**
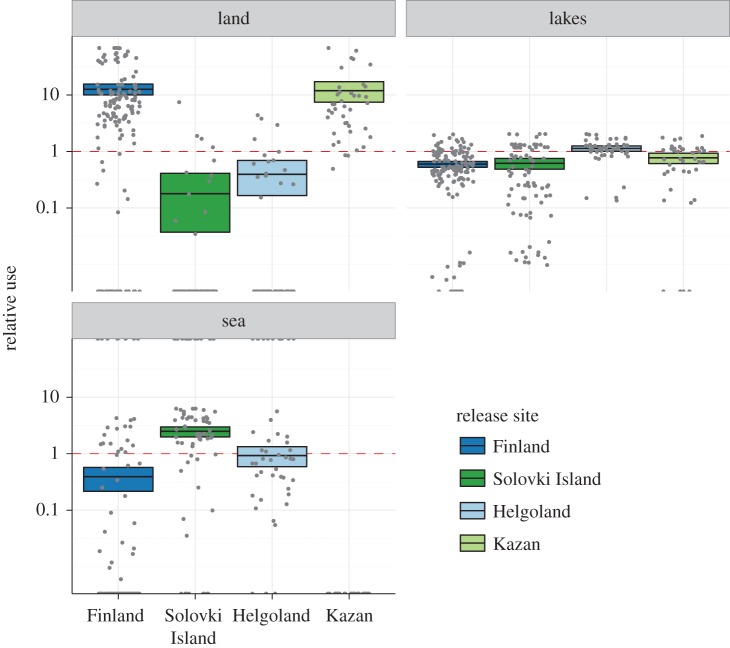
General habitat use of lesser black-backed gulls. Shown here are the relative preferences of all treatment groups for terrestrial, marine and freshwater habitats. Unbiased use of these habitat types is represented by the dashed red line. Values above the red line correspond to a positive preference (a relative use of 10 indicates that the bird was observed in a certain habitat 10 times more often than expected from the availability of this habitat type), smaller values correspond to a negative preference. Coloured boxes present the 95% CIs on the mean per treatment group (acquired through 1000-fold bootstrapping), the black bar represents the observed mean, and grey dots represent the raw data.

Dividing available habitat into three classes (terrestrial, marine and freshwater), we found that individuals from all groups differed in how intensively those three different biomes were used (figure 3). The Finnish individuals were located over terrestrial habitat 12.6 times more often than expected from the availability in the occupied area. By contrast, individuals from Solovki Island were located preferentially above the White Sea (57% of the fixes, 2.5 times more often than expected). After translocation, the use of general habitat differed from the control population. Individuals in Kazan were mostly above land (47% of the locations, 11.87 times more often than expected), whereas individuals in Helgoland were mostly associated with lakes (77% of the locations, 1.13 times more often than expected). This latter observation is caused by individuals dispersing from the island and also using mainland areas (see figure 1 and electronic supplementary material, figure S1 in the appendix).

### 3.3. Habitat models and comparison

The different MaxEnt models we computed showed high performance for both the training and the test dataset. Prediction success for the test locations (25% of the locations omitted prior to model training) was in no case less than AUC = 0.94 (test data, mean AUC = 0.975 ± 0.02 s.d.) for the group-level models. Moreover, the models showed a good performance in distinguishing between used and background habitat, as model gain indicated, exceeding 1.93 for all groups (test data, mean =3.405 ± 0.96 s.d.). Thus, the predicted probability of occurrence for actual occurrence points was at least 6.9 times higher than for random background points. Out of the initial 75 environmental layers, only a subset contributed to the MaxEnt models and were thus kept for the final models (control: 36, Helgoland: 29, Kazan: 27). The contributions of variables to the final models are listed in the electronic supplementary material, table S1.

**Figure 4.**
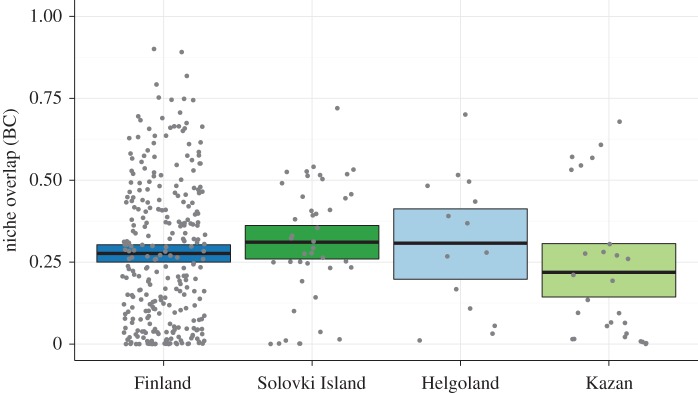
Within-group niche overlap between individuals. The niche overlap was calculated between each combination of individuals per group. Coloured boxes present the 95% CIs on the mean per treatment group (acquired through 1000-fold bootstrapping), the black bar represents the observed mean, and grey dots represent the raw data. The respective number of individuals are: Finland: *n* = 33, Solovki Island: *n* = 11, Helgoland: *n* = 9, Kazan: *n* = 8.

We found that individuals within groups differed substantially in their habitat use, which was indicated by the low overlap between models based on the locations of single individuals (Finland: BC = 0.28 ± 0.22, Solovki Island: BC = 0.31 ± 0.18, Helgoland: BC = 0.31 ± 0.21, Kazan: BC = 0.22 ± 0.22 (mean ± s.d.), see also figure 4). The amount of overlap between individuals did, however, not differ between the respective groups (two-sample *t*-tests, Bonferroni-corrected *p* > 0.15 for all comparisons (Finland–Solovki Island, Finland–Helgoland and Solovki Island–Kazan)).

Control individuals from Finland and the White Sea did not seem to occupy similar habitat, as the space use predicted by the corresponding models differed substantially, indicating ecological divergence in the two populations. The niche identity test confirmed that habitat use of the two control groups was not identical (BC = 0.215, *p* < 0.001). This difference was not solely owing to a differential composition of the habitat available to individuals in Finland and at the White Sea, as was confirmed by the background test (*p* < 0.001).

**Figure 5.**
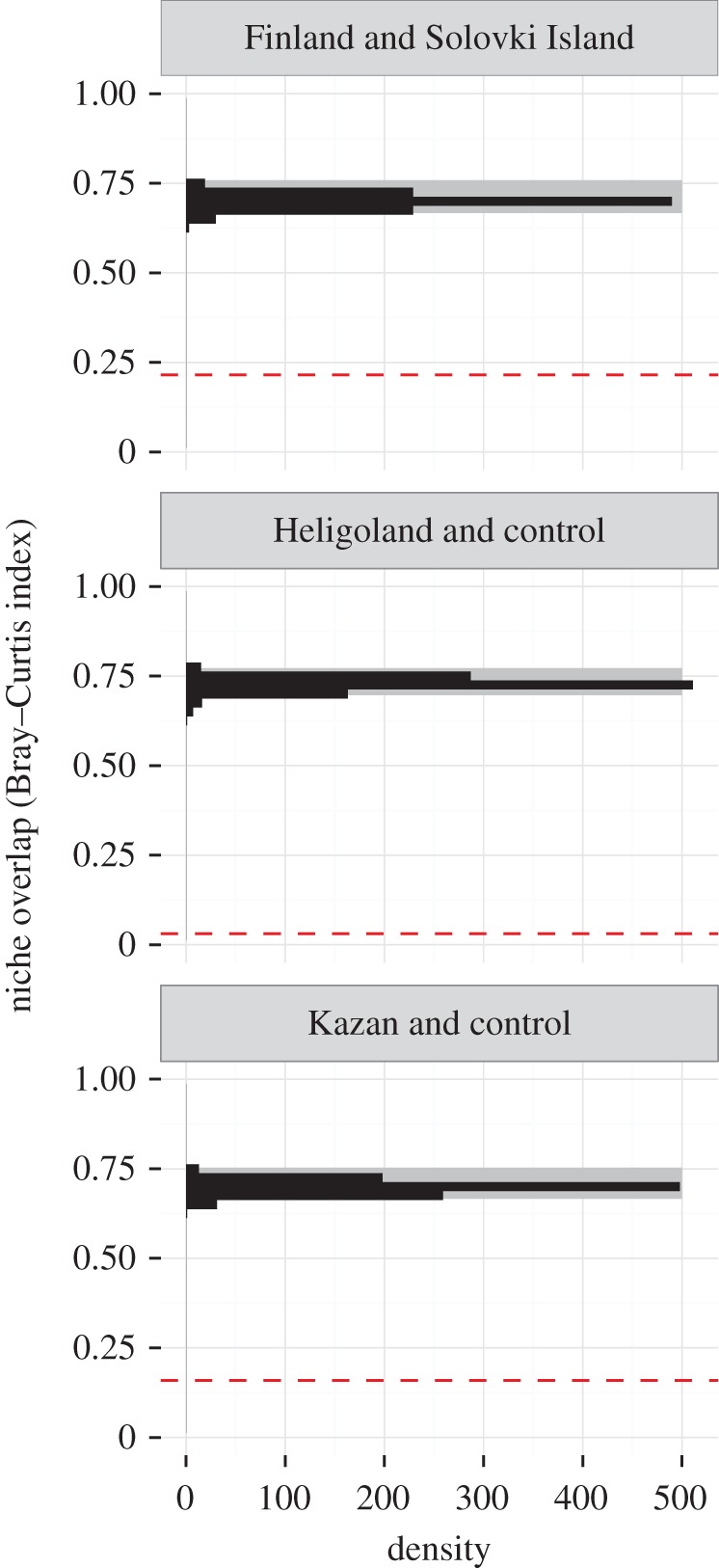
Results for the niche identity test. The dashed red line shows the observed niche overlap; the histogram represents the expected niche overlap determined by the randomizations. The grey rectangle shows the upper 95% of the distribution.

**Figure 6.**
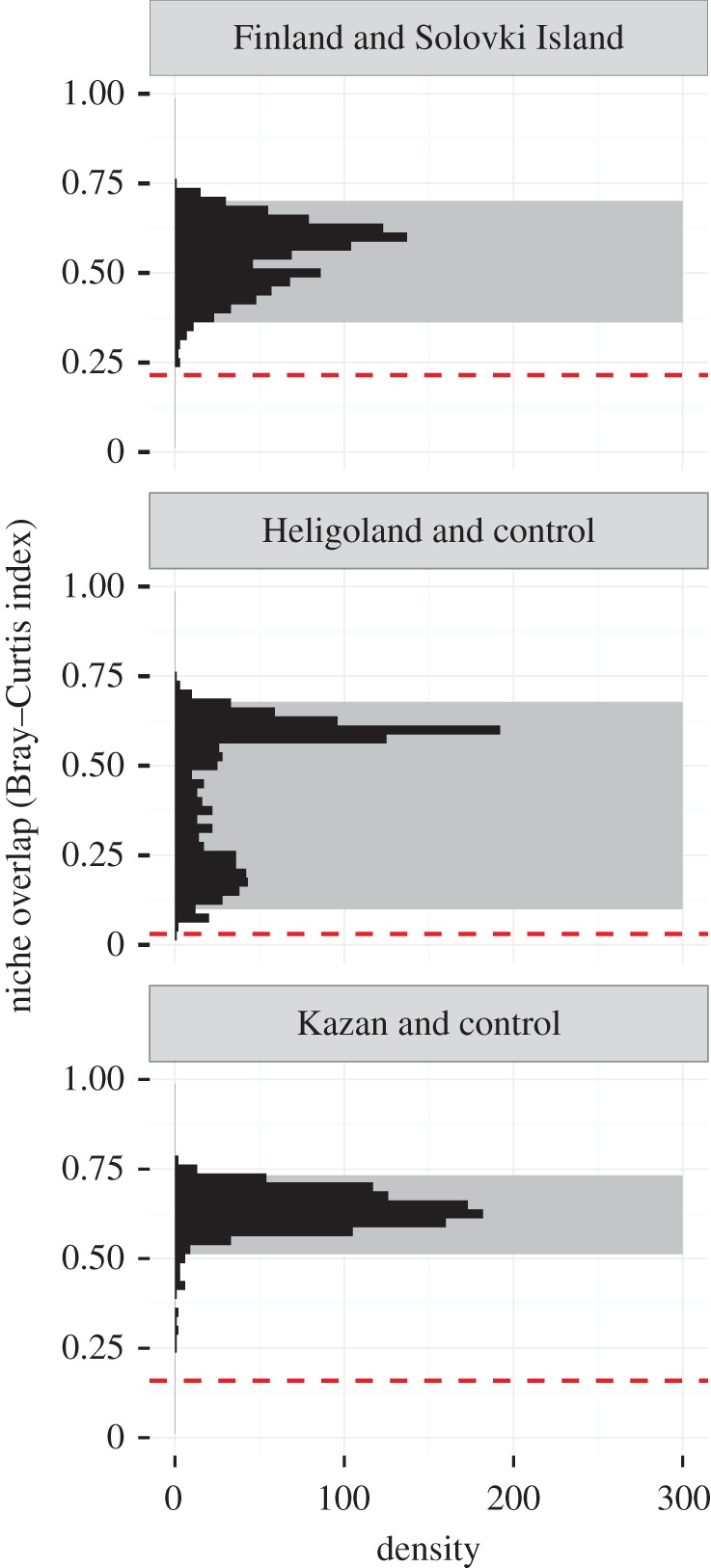
Results for the background test. The dashed red line shows the observed niche overlap, the histogram represents the expected niche overlap determined by the randomizations. The grey rectangle marks the 2.5% and 97.5% quantiles of the distribution.

Comparing the predicted space use of the translocated individuals to that of the combined set control individuals, we found no transferability. Neither within (control-Helgoland, BC = 0.030, niche identity test: *p* < 0.001, figure 5), nor outside the native breeding range of *L. fuscus* (control-Kazan, BC = 0.159, niche identity test: *p* < 0.001, figure 5) was space use well predicted by the control model. Again, these divergences of the realized niches could not be explained by differing environmental composition between the areas used by control and translocated individuals (background test, control-Helgoland: *p* < 0.001, control-Kazan: *p* = 0.001, see also figure 6).

## 4. Conclusion

Within the limits of the available data, our results show that there are considerable differences in habitat use between both treatment groups. These results suggest that individual gulls at each site readily use different habitats and associate with the local environment in different ways. When comparing habitat use between the control group and the translocated individuals, we found evidence supporting a high flexibility of habitat use that seems to be interacting with local conditions. The fact that the translocation resulted in yet different niche models compared to the most general model based on both native populations suggests that translocated individuals change the way they interact with resources immediately after the release into novel environments. Also these shifts were, according to the background tests we performed, not the mere result of the differences in the environmental conditions but rather a result of translocated individuals associating in novel and unpredicted ways with the environment. Moreover, we observed differences in habitat use between individuals in the native populations of Finland and on Solovki Island. While in isolation, the differences between individuals at the same site support results from previous studies showing consistent individual differences [21–24], the overall results are conducive of high ecological flexibility. We think that this high degree of ecological generalism at the species level contributed to the recent range expansion of lesser black-backed gulls.

Individuals in southern Finland seemed to have a preference for terrestrial habitats, whereas birds from Solovki Island had a higher preference for marine habitat (figure 3). This differential use was also reflected in the results from the niche identity and background test, suggesting that habitat use in a shared environment would differ strongly between these two populations (figures 5 and 6). If it was not for the additional translocation experiment, these findings could be interpreted as some degree of local adaptation. However, there were also clear differences in habitat use between control and translocated birds, both with respect to the use of lakes, marine and terrestrial habitat, and as indicated by the niche comparisons. Although we consider the chances that the individuals selected for translocation happened to be a non-representative subset of the original populations in both cases as unlikely, we cannot ultimately exclude that these group-level differences might have been driven by the specialization at the individual level. Within the limits of our data, however, we think that our results are a clear indication of high flexibility of habitat use in *L. f. fuscus* on a population level. This could further, and more fundamentally, be tested by studying the habitat use of individuals from the two control populations in their native habitat, and translocating them to the respective other control population and back.

An alternative explanation for the differences in habitat use between the control and translocated individuals is the difference in treatment, as translocations have been shown to induce stress and altered behaviour after release [51] that recedes on the scale of weeks [52]. However, in a previous study conducted with the same tracking data Wikelski *et al.* [34] have shown that the survival rates did not differ between treatment groups neither during the post-release phase nor during the subsequent migration. Moreover, the displacement from the release site shows that translocated birds settled quickly, albeit farther from the release site than control individuals, and initiated a regular migration to the wintering site of the subspecies in East Africa (figure 1). With breeding and natal dispersal of distances up to 200 km [53], individuals might be frequently faced with unknown areas, and we thus think that potential stress from the translocation treatment has had no decisive effect on the overall results. Another potential source of impact is the presence of conspecifics at the release site on Helgoland, where the neighbouring subspecies *L. f. intermedius* occurs. In recent years, these individuals seem to have adopted a similar habitat use as we observed for the individuals released in Helgoland [11]. We cannot exclude any influence that local birds might have exerted on the individuals released there.

Overall, we suggest that our results do not support the hypothesis of populations being adapted to the conditions locally available to them in this subspecies. We rather think that these results suggest a high amount of flexibility in exploiting different habitats. Our results are in-line with a lack of clear genetic divergence in the northern taxa of the *Larus argentatus-fuscus-cachinnans* group and support the hypothesis of a rapid spread across the Palaearctic [15, 25], as generalist species are usually characterized by the colonization of a wide range of environments. These taxa have been very successful in conquering new habitats (see also [12, 13]), and the overall population size of the species has been increasing over several decades [54, 55]. As indicated by findings from comparing the success of invasions by birds species [5], we think that in this species flexibility might be an adaptive trait in a phase of rapid expansion and population growth. Overall, we think that the approach we used is also a valuable tool to test for potential contributions of local adaptation to species divergence in systems like this species complex.

The data available to us were limited in that our main analysis could only be performed on terrestrial locations. Furthermore, the results would have benefited from an additional translocation experiment between the two control populations to understand habitat use of Finnish and White Sea individuals within the same environment. Yet, the results we presented in this study were clear enough to indicate a high flexibility of habitat use in this species (see also [11]). Using data from other subspecies, like *L. f. heuglini* in the contact zone with *L. f. fuscus* can shed further light on how the interactions between the two subspecies might change the dynamics of individual specialization. In addition, using animal observations from databases like GBIF, or experimentally exchanging tagged individuals between populations might be useful to study the potential influence of local birds at the release sites on the habitat use of the translocated individuals. More fundamentally, we show that in a flexible species like these gulls the use of just a local subset to model habitat use, and extrapolating predictions of suitable habitat, is very likely to provide uninformative results. Even in more specialized species, habitat use observed in one area might not necessarily be transferable to other locations, especially in cases where local adaptations occur. Moreover, individual specializations might further bias predictions made from habitat use of just parts of the population (see also [56]). When models of habitat use are incorporated into conservation planning it might be critical to correct for the local availability of resources, as well as potential intraspecific differences or great ecological flexibility in resource selection functions [57, 58].

## Supplementary Material

Appendix - Here we provide two additional figures showing both the tracking data that was used for modelling habitat use with Maxent and the respective spatial predictions.

## Data accessibility

Original tracking data are available from the Movebank Data Repository (http://www.datarepository.movebank.org) under (http://dx.doi.org/10.5441/001/1.q986rc29) [35].
