# Supplementary material for: Flexibility of habitat use in novel environments: insights from a translocation experiment with lesser black-backed gulls
Source: R Soc Open Sci. 2017 Jan 18;4(1):160164. doi: 10.1098/rsos.160164 (PMC5319309; doi:10.1098/rsos.160164)
Supplement: Appendix - Here we provide two additional figures showing both the tracking data that was used for modelling habitat use with Maxent and the respective spatial predictions. [file rsos160164supp1.pdf]

Flexibility of habitat use in novel environments: Insights from a translocation experiment with Lesser Black-backed Gulls.

Mariëlle L. van Toor, Elena Arriero, Richard A. Holland, Markku J. Huttunen, Risto Juvaste, Inge Müller, Kasper Thorup, Martin Wikelski & Kamran Safi

Supporting Information

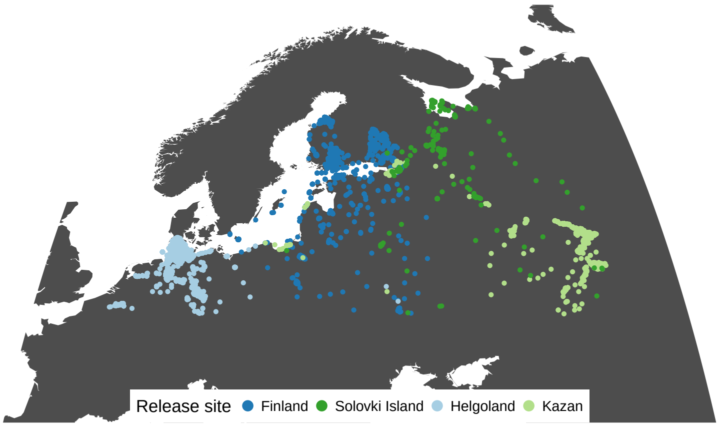

Figure S 1. Tracking data of Lesser Black-backed Gulls. The data shown are the locations used for modelling.

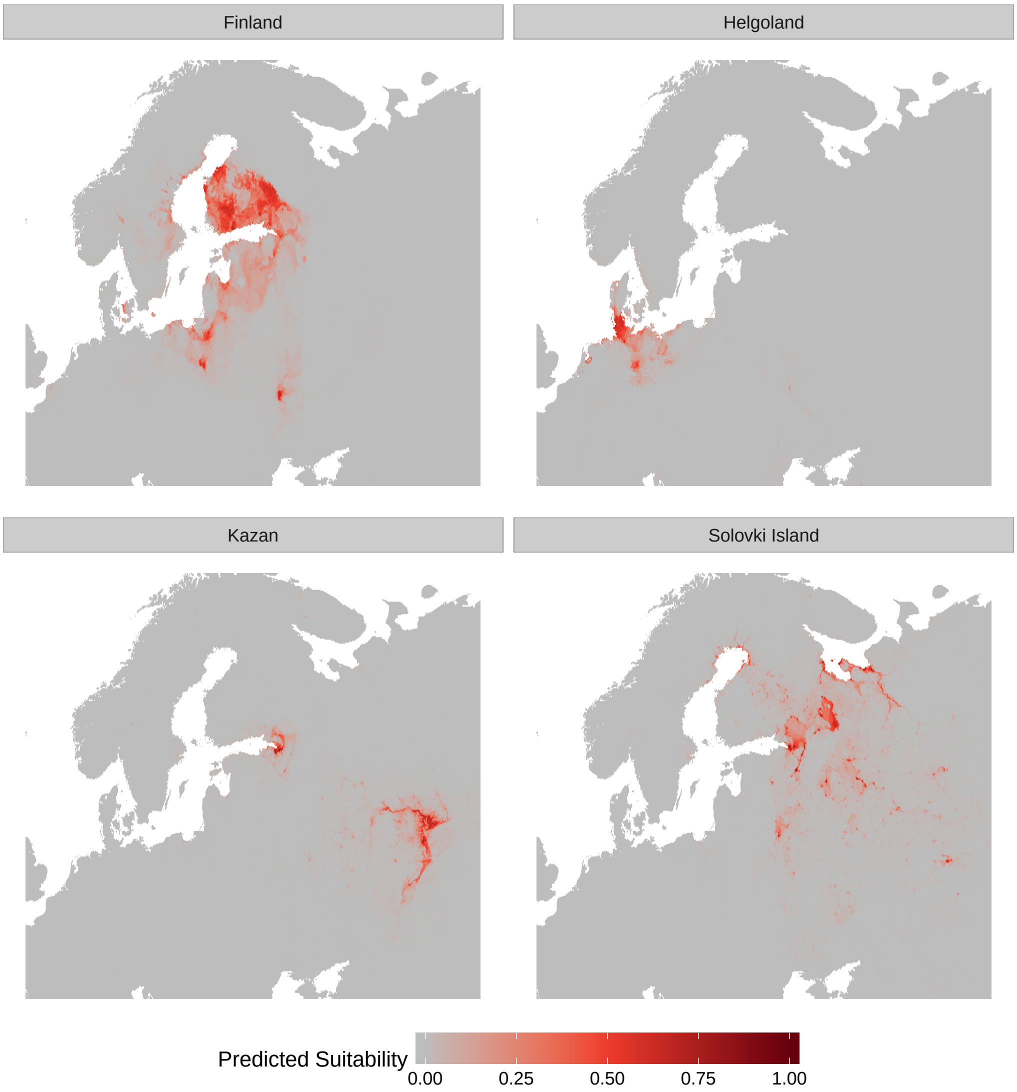

Figure S 2. Spatial estimates of habitat suitability for both the control and translocated groups as predicted by the species distribution models. Grey areas designate unsuitable habitat, red areas are as or more suitable than the minimum suitability predicted for actual presence locations of the gulls.

rsos.royalsocietypublishing.org R. Soc. open sci. 4: 160164

Table S 1. Contribution of variables to the final MaxEnt models. Here we list the variables that contributed to the final models for a) the control individuals, b) the individuals released on Helgoland, and c) the birds transported to Kazan. Shown are all variables with a contribution greater than 1%.

Variable name	Percent contribution to the MaxEnt model
**a) Control group**	
Altitude above sealevel	16.8
Precipitation (May)	13.6
Bioclim 4	10.7
Terrestrial ecoregions	9.9
Global Lakes and Wetlands	7.5
Precipitation (August)	6.3
Bioclim 15	6
Precipitation (September)	5.8
Minimum temperature (October)	4.6
Precipitation (November)	3.8
GlobCover_2009	3
Maximum temperature (July)	2.1
**b) Helgoland**	
Altitude above sealevel	16
Minimum temperature (March)	15
Minimum temperature (January)	14.5
Minimum temperature (November)	14.3
Maximum temperature (October)	7.9
Precipitation (December)	7.3
Precipitation (November)	4.6
Precipitation (September)	3.8
Bioclim 7	3.5
Precipitation (August)	2.7
Precipitation (May)	2.1
Bioclim 6	1.7
Terrestrial ecoregions	1.7
**c) Kazan**	
Distance to sea	26.4
Terrestrial ecoregions	23.8
Altitude above sealevel	14.4
GlobCover_2009	10.8
Bioclim 9	7.7
Bioclim 15	3.8
Precipitation (September)	3.5
Precipitation (August)	1.7
